# Does Gallery Lighting Really Have an Impact on Appreciation of Art? An Ecologically Valid Study of Lighting Changes and the Assessment and Emotional Experience With Representational and Abstract Paintings

**DOI:** 10.3389/fpsyg.2019.02148

**Published:** 2019-10-04

**Authors:** Matthew Pelowski, Andrea Graser, Eva Specker, Michael Forster, Josefine von Hinüber, Helmut Leder

**Affiliations:** ^1^Empirical Visual Aesthetics Lab, Faculty of Psychology, Department of Basic Psychological Research and Research Methods, University of Vienna, Vienna, Austria; ^2^Studio Okular, Vienna, Austria

**Keywords:** lighting, art perception, context, ecologically valid, gallery, aesthetic emotion

## Abstract

We report two studies considering the potential for gallery lighting conditions to modulate appraisals and emotional experience with works of visual art. As recently documented in a number of papers, art appreciation represents a complex blend of formal artwork factors, personalities and backgrounds of viewers, and multiple aspects of context regarding where and how art is experienced. Among the latter, lighting would be expected to play a fundamental role. However, surprisingly, this has received little empirical assessment, with almost no ecologically valid gallery analyses and no between-participant designs which would minimize awareness of lighting changes themselves. Here, we employed a controlled paradigm using a spontaneous art viewing context, a gallery-like setting, and a proprietary lighting system which allowed the minute adjustment of lighting intensity/temperature (CCT). Participants viewed a selection of original representational and abstract art under three different CCT conditions (Study 1), modulated between participants, and then reported on their artwork appraisal and emotional experience. The selected lighting temperatures were chosen based on an initial investigation of existing art museums within the Vienna area, addressing how these institutions themselves light their art—a question which, also somewhat surprisingly, has not often been considered. We also allowed the same participants to set the light temperature themselves in order to test hypotheses regarding what might be an ‘ideal’ lighting condition for art. In Study 2, we explored the question of whether artworks made by an artist to match specific lighting conditions show a resulting connection to the ratings of viewers when shown in the same or different light. Results showed almost no effects from lighting changes in both studies. Viewers’ self-set light temperature (mean = 3777 K) did roughly coincide with the suggested most enjoyable conditions for everyday living and some past research on art viewing, but again showed wide interpersonal variance. Results, and a general review of lighting factors are considered in order to provide art researchers and curators with a tool for conducting future study.

## Introduction

Art experience is a complex activity. Engaging art can involve numerous processes, from meaning-making to emotions and appraisals, to personal associations and body response (e.g., Leder et al., 2004; Pelowski et al., 2016), all of which might blend together to produce an aesthetic experience. Even more, emerging research has also highlighted the fundamental role of context in modulating how art is reacted to and appreciated. When we do approach an artwork—in our homes, in a laboratory, and perhaps most saliently in a gallery or in a museum—our interaction is made under the influence of a wide range of factors—setting, hanging conditions, expectations, other people (Newhouse, 2005; Pelowski et al., 2017a for review)—that can color or even change our experience.

One factor that—intuitively—would be expected to play a fundamental role in art perception, is the lighting of art itself. As a visual species, and certainly since the inception of civilization, lighting has been a key aspect of human life (Werth et al., 2013). Lighting may spotlight and guide our attention. It may provide a tone or mood to our environments. Lighting may also be a key aesthetic aspect for artists. Both in art production and in final artwork reception, lighting may interact with certain colors or materials, and be a key part of the ambient art making-(such as North facing studios or plein air painting) environment. The use of lights to highlight and often to enhance artworks is also a universal practice in museums. Each individual museum may spend a great deal of money and attention on lighting, to very different effect. This goes hand-in-hand with an increasing variety of lighting technologies (e.g., LEDs, which can reduce issues of damaging ultraviolet or infrared radiation that had limited previous lighting options), providing curators a wide pallet of light intensities or color temperatures (e.g., Pridmore, 2017) and leading to arguments (e.g., Druzik and Eshøj, 2007) that lighting is *the* most complex and, thus, one of the most important factors in museum design, combining technology with perception, cognition, appreciation, and psychological experience.

However, perhaps due to the very same issues of multiple lighting varieties, potential modulating factors, and difficulties in access to museum spaces and in the ability to change lights, there is little systematic artwork lighting research. It is not established, for example, if there is an ‘ideal’ lighting condition for art objects. Nor are there standardized procedures for systematic study designs or controlled investigations focused on artwork enjoyment (Scuello et al., 2004b; Michalski, 2007; Nascimento and Masuda, 2014). Equally important, there is a need for empirical research that focuses on the actual impact of different lighting conditions on the spontaneous, ecologically valid experience with art. Present studies, which most often come from technically- or lighting-focused rather than art-focused perspectives, have almost exclusively used lab reproductions (e.g., light boxes with miniature art dioramas or screen-based images with computer generated lighting) and within-participant designs that ask individuals to make multiple appraisals of the same art object—typically assessing simple preference for lighting combinations—with light adjustments themselves very salient. We do not yet know if these results lead to important differences from actual gallery interactions, nor how lighting might impact a wider range of appraisals or emotional and even economic reactions. Nor do we know whether lighting changes, if obscured from the viewer as merely part of the overall museum engagement, have *any* measurable impact, leading to a glaring omission in present museum art research.

This paper offers a first between-participant analysis of the impact of lighting on the appreciation of art as this manifests in ratings, economic decisions, and emotional experience. This was done using an ecologically valid spontaneous art viewing gallery context and the use of a lighting system which allowed the minute adjustment of lighting intensity and temperature within the space, in conjunction with original representational (Study 1) and abstract (Study 2) paintings and a with lighting conditions modulated to minimize awareness of the actual lighting itself. The selected lighting temperatures were chosen based on an initial investigation of existing art museums within the Vienna area, addressing how they themselves light their art—a question which, somewhat surprisingly, has itself not often been considered (Kesner, 1997). In Study 2, via a unique opportunity to work with the artist of our study materials, we also explored the question of whether artworks made by an artist to match specific lighting show a resulting connection to the actual ratings of individuals when shown in the same or different lighting conditions. Because this paper is aimed at the researcher interested in the perception of art, whereas most previous literature is currently in the domain of commercial or technical lighting research, we also begin with a review of main theoretical and practical aspects of lighting choices and existing empirical studies for use in framing this and future research.

## Review: Lighting of Art, Key Factors, and Past Research

In order to contextualize the following studies, it is first useful to consider: (1) what are the main parameters of lighting, how do these vary or correspond to technologies (i.e., bulbs or lighting systems), and how do these connect with curator decisions? (2) What are the existing parameters currently applied to art within galleries? (3) What is the existing art-related research from which this study can build and outstanding questions?

### Main Lighting Factors and Types

When curators and museums approach lighting, there are of course several, potentially dueling, factors that might be considered (Scuello et al., 2004b for review). These include conservation and protection of art. Light—especially with paintings or other delicate materials, and with traditional incandescent or gas discharge fluorescent lamps which may emit infrared or ultraviolet light, as well as natural daylight—can cause photochemical damage leading to fading, yellowing, etc. (Fördergemeinschaft Gutes Licht, 2000). Thus, much of the earliest lighting research in museums focused on the conservation aspect, examining the effect of light on material (for review see Nascimento and Masuda, 2014; Pridmore, 2017) or providing suggestions for best practice so as to protect art (e.g.,Commission Internationale de l’Eclairage [CIE], 2004, see also below).

At the same time, emphasis is also given to “aesthetic” (Scuello et al., 2004b, p. 306) aspects of lighting choices. Museums and curators of course want to showcase their art and their spaces in the ‘best light’ and/or to provide an optimal viewing experience. Here as well, several factors may be important: for example, the brightness or clarity of objects or of details and light’s general color (Linhares et al., 2009; Nascimento and Masuda, 2014), reflections of lights from object surfaces (Cuttle, 2007; Druzik and Eshøj, 2007), contrast, ability of lighting to reveal brushstrokes or textures, diversity of illuminated colors (Pinto et al., 2008; Nascimento and Masuda, 2014; Pridmore, 2017), as well as to provide a certain mood to a gallery or to generally increase comfort of viewers (Feltrin et al., 2017). Among these, and when describing art lighting choices, two technical parameters are however most commonly considered: (1) general brightness or illuminance and (2) color temperature.

Brightness, or more precisely, lighting intensity, is defined as the proportion of light that falls on a unit of area. This is typically denoted by the measure of Lux (‘lx,’ luminous energy by unit time, indicated in lumens per the surface area in square meters)^1^. Color temperature provides a means of quantifying the color impression of a light source (Paul, 1999). This is usually expressed as the “correlated color temperature” (CCT), measured in Kelvin (‘K’), and denoting the temperature at which a blackbody radiator has the same color appearance as a source of light. This is a function of the specific spectrum of wavelengths making up a light, with relatively longer wavelengths seen as more yellow/orange and reddish, and shorter wavelengths more blue/purple. The specific balance of wavelengths and their respective power is then perceived by the viewer as a shade of color (Kienle, 1941), often subjectively described as relatively more “cold” (blueish light, having higher power among shorter wavelengths, but, somewhat counterintuitively, of relatively ‘higher’ Kelvins) or “warm” (higher power among reds and oranges of longer wavelengths, but of a lower Kelvin measure).

**FIGURE 1:**
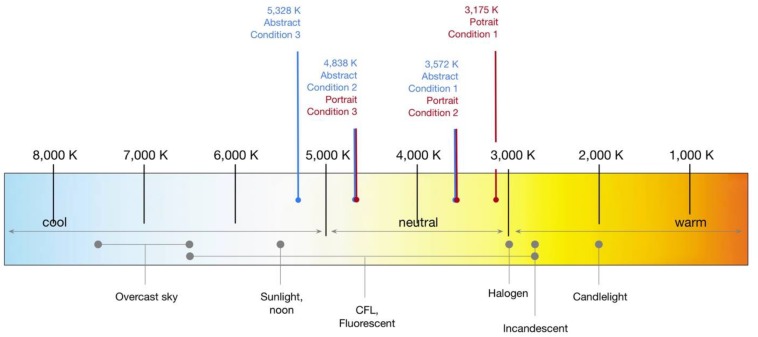
General range of visible light temperatures and specific lighting conditions from Art Museums used in Studies 1–2 (relative temperatures of light technologies are suggestive; see e.g., Paschotta, 2008) (All figures created by the authors).

As shown in Figure 1, which displays several common light bulb types on a spectrum as well as the specific lighting conditions used in our forthcoming studies, artificial lighting styles tend to range from candlelight (∼2000 K) at the subjectively warmest extreme, to incandescent (2700 K) and halogen (3000) bulbs, providing a yellowish impression, to fluorescent or CFL bulbs that can have a wide range from yellow to quite blueish (3000 to 6500). Noon sunlight, about 5500 K, tends to be cooler than most indoor lighting.

Light’s color temperature also interacts with an object’s colors. Light of similar wavelength to an artwork’s colors tends to accentuate these or make them stand out, while light that has an imbalance of power at certain wavelengths tends to diminish the appearance of colors at the opposite end of the spectrum—for example, leading to blackened blues from yellow lighting. The overall color accuracy or naturalness provided by a light, as a function of its balance of spectral power, is given by the Color Rendering Index (CRI). This is derived from a comparison of the color appearance of objects under a test light source against a standard light of the same color temperature, denoted on a scale from 0 to 100 (perfect duplication; see Veitch and McColl, 2001).

As will be seen in the review below, CCT, and to a lesser extent brightness, is the main focus of most past lighting research, and argued to play a key aspect of lightings’ subjective experience or viewer preferences (Pridmore, 2017), and thus will also be the main focus of the present study.

### Typical Lighting Conditions in Art Presentations/Museums

When looking to existing lighting choices in museums, the above factors appear to arise in a general range of combinations (Scuello et al., 2004b). Until quite recently, lighting decisions and research were primarily driven by needs of conservation. The total radiant energy from a light source that makes its way to an artwork is a function of lux times wavelength—with shorter (cooler color) wavelengths thus subjecting artworks to more total energy and, over a day or a lifetime of exhibition, more potential damage (Veitch and McColl, 2001). This aspect led to suggestions for best practice, with guidelines (e.g., Commission Internationale de l’Eclairage [CIE], 2004; see also Scuello et al., 2004b; Wilson, 2006; Druzik and Eshøj, 2007) recommending keeping art exposures in the range of 50 to 200/300 lx (with the higher number often used with medium sensitivity artworks such as oil paintings) and around 3000 K CCT.

The above guidelines, here too also contained an implicit aesthetic component. The selection of color temperature in relation to brightness followed work in the mid twentieth century by Kruithof (1941; see also Scuello et al., 2004b; and Fotios, 2017). This described a curve suggesting that a preferred range of color temperatures varies with illuminance. The midpoint of the range at 200 lx was 3000 K. Although the exact suggestions from this work have been questioned in more recent empirical research (e.g., Fotios, 2017), many museums followed these guidelines, especially when they employed incandescent gas, fluorescent, and tungsten halogen lighting (Berns, 2011; Pridmore, 2017), which, as recently as a review by Pinto et al. (2008), were suggested to still be the most common modes of lighting in museums.

Note, the above ratio entirely omits color temperatures approaching open daylight—although many museums do employ baffled skylights where possible. Wilson (2006; see also Chartered Institution of Building Services Engineers [CIBSE], 1994) also suggest that a full appreciation of color itself is not possible until about 250 lx. Warm lighting also tends to desaturate blues in paintings, due to blue being complimentary to yellow, and may not accentuate more contemporary pigments of Modern and contemporary painting (Pridmore, 2017). More recent lighting technology such as LED do not emit UV and IR radiation and have a reduced visible radiation, thus potentially providing a fuller spectrum and cooler lighting temperatures (Pinto et al., 2008; Berns, 2011; Pridmore, 2017). This has led to an increasing range of options. For example, Pridmore (2017) suggests that replicated-daylight lamps in use in galleries in Europe and the United States are available in 3500, 4100, 4700, and 5000 K, and that tunable LED fixtures can range from 2000 to 5000 K.

### Previous Art/Museum Lighting Studies

Despite the above range of technological advancements and outstanding questions, actual decisions for lighting are still typically based on subjective opinions of curators or museum directors or the above rules of thumb (Druzik and Eshøj, 2007; Nascimento and Masuda, 2014; Pridmore, 2017). Empirical research on the interaction of lighting and art is only now emerging. We briefly review these approaches considering art or museums below (We have also collected the past studies, with more in-depth details on their specific methodology and design aspects in Supplementary Appendix Table A1).

As can be seen in the subheadings below and in the Table, previous research has generally followed four main varieties and essentially highlighted a lack of empirical consensus regarding approaches or lighting’s actual impact:

#### Survey/Comparison of Different Museum Conditions

The first type, and one of the earliest contemporary investigations (Kesner, 1997), used a qualitative/interview approach to compare general expectations regarding differing lighting factors among museum decision-makers (conservators, curators, exhibition designers) at multiple United States museums and asked how much effort and money should be invested toward each factor. The answers were joined by a similar survey given to visitors within museums of art. The study found, perhaps unsurprisingly, that both groups had different expectations. For museum staff, preservation was again the most important factor, and reducing glare or viewer visual comfort least important. Visitors gave particular emphasis to art appearance (color range, attractiveness; as might relate to especially CCT), but, interestingly, also claimed that brightness/contrast and reducing glare were least important. Also interesting, following the initial Kesner study, this type of comprehensive documentation of museum conditions is quite rare. Wilson (2006) briefly reported a similar project to measure the relation between background, lighting, and artifacts (mostly stone sculptures) in archeological museums, matched to visitor appraisals of the lighting. Lighting quality was primarily judged—in this case—by degree of contrast between object and background, but again not brightness. They noted that in some visitor-preferred configurations detail or clarity of the object would be lost, but, “visitors are unaware of this loss.”

#### Self-Selection of Preferred Lighting

Second, empirical investigations have more directly tested lighting by asking individuals to either self-select certain lighting parameters (typically CCT and in some cases brightness or CRI) according to their taste or by using a series of forced-choice art/lighting comparisons.

Scuello et al. (2004b) displayed postcard-sized reproductions of impressionist/representational paintings (four total, divided among differing main colors, i.e., blues or reds) in light boxes. Viewers (*N* = 9) could look into the box through a porthole as if it was a diorama of a gallery. Using a forced-choice design, for each trial viewers looked at the same individual painting in two different dioramas with two (of eleven total) light conditions varied in CCT (2500–7000 K; illumination intensity kept constant at 200–250 lx) and selected “whether the painting looked better” and by how much using a 6-point scale. This was repeated for all paintings/illumination combinations, leading to each painting being seen 20 times under each given illuminant. The results suggested that 3600 K—generally supporting the previous rules of thumb above—had the highest percent of cases where it was selected against the alternative. However, the authors note, the results were “not overwhelming.” A second spike of CCT preference also occurred at about 5400 K, whereas 3200 and 5000 K showed generally lowest preference. There were also pronounced individual differences, which were not further investigated. They also found very minor evidence for a potential interaction between paintings and lighting. Assessing the art after first acclimating oneself to differing CCT/illumination combinations, in an attempt to replicate what might occur when an individual enters a gallery from a different room, had no effect (see also Scuello et al., 2004a and Supplementary Appendix Table A1, for a follow-up study with similar, highly variant, findings).

Another set of related studies, first by Pinto et al. (2006), collected hyperspectral images of five Renaissance era oil paintings (all Madonna and child with dark background). These were used to create computer-generated images reproducing the art’s appearance under five CCTs (illumination 200–400). Participants (*N* = 5) viewed the paintings on a monitor in the lab, with a trial involving the same painting shown twice in sequence with two different lightings and participants asked to choose which they preferred. Each lighting-art pair was observed 20 times (total 500 trials; 100 evaluations made for each individual painting). Across all but one painting-lighting combination, participants preferred higher color temperature (4450–6500), although it was unclear if this was simply a contrast effect tied to light rather than the interaction with art.

Pinto et al. (2008) conducted an extension with 11 oil paintings from the same museum, computer-generated to appear under CCTs from 3600 to 25,000 K (21 equally spaced steps), and a much larger sample of participants (*N* = 80), divided between art-novice undergraduate students (participating in a laboratory) and art museum visitors (using a computer to participate within the course of their visit to the museum). The lab participants assessed each painting three times on different days; the museum visitors once. Generally, the most preferred CCT was a cooler 5100 K, although again different peaks were found for each painting and participant.

Nascimento and Masuda (2014) conducted a similar study but with the real examples of the above artworks. Participants first viewed each painting on a monitor as above, selecting the ideal CCT (3600–20,000 K; 200 lx), with the entire painting set shown twice in random order. This was followed by a short break and the viewing of an actual paintings, hung on a wall, each shown individually with adjustable lighting. The entire task was repeated with a total number of ratings for each artwork in monitor condition of 8; real condition was 12. In order to ensure an even coverage of light in the real condition, the paintings were partially covered with a black frame. The study used seven participants. No mention was made of whether participants stood in the real art condition, nor if this approximated in any way an actual gallery. Both conditions returned similar results, with an average CCT of 5500 for real, 5700 K for monitor, but once again with a CCT varying between paintings—with roughly half having a mean CCT lower than the previous study’s finding and half higher—and also varied markedly between participants.

#### Scale-Based Rating of Different Lighting-Art Combinations

Third, a few studies have begun to move beyond basic preference to a broader set of scale-based ratings, however still focusing on general artwork appearance. Luo et al. (2013) used six hand-painted copies (made by other artists) of original pieces from the Taipei Fine Arts Museum. The artworks were illuminated by 15 CCT/illumination combinations and placed in a light cabinet (no information given on hanging aspects). Participants (30; half science/engineering students, half art students) sat in front of the cabinet and viewed the art in each lighting combination and rated the paintings for physical attributes (colorful/dull, bright/dark, clear/blurry) and for “psychological perception” factors (warm/cold, relaxed/tense, soft/hard, natural/unnatural, active/passive, comfortable/uncomfortable, modern/classical, pleasant/unpleasant). The preferred lighting was 5000 K for art students and 4000 K for science students, both at 300 lx. Principal Component Analysis of the appraisal scales also suggested two components— “warmth” (warm/cool, classical/modern, soft/hard; presumably connected to CCT) and “visibility” (all other scales, including pleasantness).

Feltrin et al. (2017) affixed a painting on a metal stand at a typical hanging height with a viewer seated in a chair looking into the space (similar to Scuello et al., 2004b), and viewing one of five reproductions of impressionist paintings printed on canvas and with either a prominent color of red, blue, green, yellow, or their combination. The pictures were illuminated with five CCTs (all ∼160 lx) and backed with three different curtains (white, gray, black) to test interaction of lighting, art, and background color. Participants (25, with nine fellow researchers in the same laboratory) viewed each painting under all lighting conditions shown successively in random order (totaling 15 viewings per painting) and reported assessments using six bipolar scales (painting color’s warmth, vividness, brightness, attractiveness; as well as overall appreciation of the arrangement and of the background). Importantly, with each new painting, all five light configurations were first cycled through in order to give participants an idea of the differences, but of course also making the changes very salient. Results, as is of course a theme throughout this review, showed a range of preferences, with CCTs of 3500, 4000, and 5000 K nearly equally preferred. Background color again showed no difference. A similar preference trend was also found for all paintings regardless of the predominant artwork hue (again generally similar to Scuello et al., 2004b).

#### Lighting Studies in Original or Approximated Gallery Settings

Finally, to our knowledge, only three studies have actually considered art as it might be encountered by a viewer moving somewhat naturally inside a gallery setting. Balocco et al. (2018) used a room with a wall-sized fresco depicting a tree-lined path with a building in the background, and gardens and architectural elements in the foreground. The artwork was lit with three accent lighting conditions modulating CCT. Participants (*N* = 15) were asked to enter the room and perceive the artwork as it was lit in succession by all three configurations for 15 s each, “expressing [their] own preference.” Participant then selected which light they preferred, with the entire paradigm repeated three times (no mention of balancing/randomization). They also employed mobile eye-tracking to consider impact of the light on looking patterns. Roughly half (54%) of participants preferred the coolest (4049 K) light. Participants also showed generally similar areas of visual interest and visual pathways across conditions. However, the preferred, bluish light had relatively more fixations on areas of interest before moving to another, and lower transition entropy, which they suggest might tie to higher clarity of colors and brightness.

Yoshizawa et al. (2013; see Zhai et al., 2015 for results and discussion) provided a brief report in a conference proceeding of one of the most ambitious approaches. They first employed a mockup gallery space with reproductions of three oil paintings (16th century portrait, 19th century impressionist landscape, 20th century abstract) under 52 combinations of CCTs, illuminances, and CRIs. They also conducted a second study in the Morohashi Museum of Modern Art in Japan with real oil paintings seen under nine CCT conditions (illuminance constant). Participants (number not known) viewed each painting-lighting combination and made evaluations using bipolar scales. The ratings were assessed with Structural Equation Models. These suggested the two factors of “visibility” and “texture” were most important for driving preference in both conditions. Notably, while illuminance did show some importance for determining subjective assessment of both factors, CCT showed the strongest relation, with a negative correlation regarding texture and a positive correlation with visibility. Color rendering showed only very low relation to preference variance.

Zhai et al. (2015) conducted a similar study on the combined impact of CCT and illuminance on art appearance and on the general mood or felt “atmosphere” of a gallery. They employed a room mocked up to resemble a museum gallery (white walls, wood flooring), with six paintings (all representational with a slightly impressionistic style; using similar muted pinks, oranges, and blues). These were hung, individually, on one wall. A LED was used to illuminate the paintings, notably acting as a directed spotlight rather than lighting the entire room evenly, and with 12 combinations of CCT/illuminance, corresponding to the lower and upward limits of recommended lighting in museums (Commission Internationale de l’Eclairage [CIE], 2004). Participants (*N* = 24, divided equally into students majoring in non-art and art fields) viewed each of the paintings under all of the lighting conditions, shown in succession, with the viewer making a rating for each combination using six scales expected to relate to “appearance” (Warm/Cool, Bright/Dark, Clear/Unclear, Colorful/Dull, Natural/Artificial) and eight scales relating to “atmosphere” (High/Low Quality, Active/Negative, Relaxed/Tense, Soft/Hard, Artistic/Business, Lively/Boring, Comfortable/Uncomfortable, Pleasant/Unpleasant). No mention was made of whether the relation of the scales to the paintings or to the room atmosphere was actually communicated to participants. A principle component analysis and Structural Equation Model returned components involving clarity, warmth, brightness, contrast, comfort/pleasantness, and finally “artistic aspects” (relaxed, warm, soft, artistic). Especially, ratings for this latter group decreased as CCT increased (becoming cooler). On the other hand, ratings for contrast, brightness, clarity, and quality showed an opposite pattern. They also suggested that the results “implie[d] that different paintings could be enhanced by applying different lighting conditions,” although they do not discuss these differences.

### Summary, Issues, and Outstanding Questions With Previous Art Gallery Lighting Research

Overall, the present lighting and art studies, although providing important tools and bases for study designs, do not provide clear or consistent effects. They also include methodological decisions or study foci leaving open many important questions especially for the ecologically valid art engagement. Notably, many of the studies have very small samples (e.g., less than ten) with a range of methodologies and can only be treated as purely exploratory.

Across the above studies there is a quite high variability and inconsistency with even basic aspects such as color temperature (CCT) and illumination. Beginning with CCT, a summary of the reviewed studies shows not only do they not often coincide with the beginning rule of thumb of the typical Kruithof/museum 3000 K range, these have been all over the map, often depending on the individual study: e.g., from 2850 or 2900 (Liu et al., 2013; Zhai et al., 2015) to 5500–5950 (Liu et al., 2013; Nascimento and Masuda, 2014), and notably with several studies reporting both lower and higher CCT preferences in the same analyses depending on different viewers or set-ups. At the same time, more advanced statistical models (Yoshizawa et al., 2013) suggest that CCT especially may have an important impact on subjective assessment of art. Similar variance is found for brightness, although in art ratings, rather than assessments of clarity, this does not appear to have such importance (see Yoshizawa et al., 2013 for empirical study-based argument; Kesner, 1997 for similar qualitative findings).

Importantly, in trying to unite and understand these differing findings, Nascimento and Masuda (2014) suggested that the CCT differences could be due to study design, with studies using miniaturized paintings or light boxes suggesting preferred illuminants with relatively lower CCTs (around 3600 K), whereas studies with art photographs taken from a gallery but shown on a computer monitor suggest higher preferred CCT (around 5100 K) and experiments with tunable LED on actual paintings leading to a range of CCTs from 3000 to 6000 K, depending on the painting. This argument notably also omits consideration of art in an actual gallery setting. Similar issues can also be raised for the more artwork-focused ratings, which show equally varied findings.

The above issues also raise the importance of ecological validity in regards to art viewing conditions in general. Even beyond the many existing studies conducted on a monitor, which could obviously show differences from real art engagement—i.e., relating to texture, brushstrokes, technique, highlighted differently by different lighting conditions (Pelowski et al., 2017a)—the studies that attempted to mimic a gallery space did so, in most cases, by mocking up a single wall or by only letting individuals look into the space, often with a seated viewer (e.g., Feltrin et al., 2017). By excluding the viewer in this way, this means that the individual is not actually within the lighting and not sharing the same environment as the art pieces. Use of carefully controlled light boxes, although perhaps ideal for focus on uniform lighting, could also omit important aspects. For example, Wilson (2006) suggests that diffuse illumination may increase clarity but also may omit texture on the surface of a painting.

#### Would a Between-Participant Design and Focus on the Artwork Show a Lighting Impact?

Perhaps most pressing, there is a major question regarding the within-participants designs of past studies and the nature of rating questions. Asking participants to re-rate the same paintings multiple times, in some cases more than ten or twenty ratings for the same artwork, raises serious issues for art appreciation. Although this method obviously has advantages for comparison, the study designs put very obvious stress on the subtle differences between lighting conditions, raising the question of whether it is this design that is driving most results. Most current art studies, which focus on the perception and ratings of the art itself, also stress spontaneity and use of images previously unseen and reducing repeat viewings due to conflation that can occur from previous exposures (fluency, familiarity, mere exposure, contrast effects, etc.; see e.g., Forster et al., 2013). This is also coupled with almost a complete lack of ratings meant to assess the actual enjoyment by participants of the works of art themselves. Rather, questions are almost always addressed to whether an individual prefers a certain light-artwork combination. Thus, it is interesting to assess whether participants might show differences if assessing only the art without obvious awareness of lighting, or how they might answer more hedonic or pragmatic questions of interest to curators or art researchers.

The above issues, essentially, raise the need for a between-participant design. Interestingly, the above arguments, coupled with the present lack of clear effects in studies that do tend to force awareness of, and perhaps subtle differences in, lighting, raise the rather cynical question of whether light has any impact if it were to be tested in such a way, within an ecologically valid art interaction. Veitch and McColl (2001) make this point in their review of one of the more intriguing series of studies for lighting’s impact on evaluations or performance—the experiments conducted in the 1920s at the Western Electric plant in Hawthorne, Illinois (Snow, 1927). These involved researchers changing lighting within a designated room of the plant—changing bulb types, increasing and decreasing illuminance; pretending to make changes. In every case, whatever the modulation, performance increased, suggesting only a placebo effect. Veitch and McColl (p. 8) conclude “one lesson to be learned from this series of investigations is that lighting research [may include] the confounding effect of participant expectancies, which can seriously bias empirical outcomes.” This is particularly so with within-participant designs “because the nature of the stimulus is impossible to hide [from] subjects.”

The argument for only a minor impact from lighting may also be supported in current research that has used between-participants paradigms to investigate the impact of lighting on mood or the ‘feel’ of a space. Lighting choices, much as with art, are argued to impact mood, most often following the suggestions that we may tend to feel more pleasant in warm/low-lux light and perhaps be more alert in cool/high-lux environments. However, the handful of studies that have tested lighting impact on mood changes using a between-participant paradigm have not found strong or consistent effects in both laboratory and field experiments (see e.g., Baron et al., 1992; Knez, 1995; McCloughan et al., 1999; Knez and Kers, 2000). See also Boray et al. (1989), who reported no differences in attractiveness ratings of human actors between three CCT conditions.

More generally, studies on visual perception also support the suggestion that individuals may be quite good at minimizing lighting impact. The so-called ‘color constancy phenomenon’ (Foster, 2011; see Nascimento and Masuda, 2014; Berns, 2016; Pridmore, 2017 for discussion in context of art) suggests that human vision tends to maintain the impression of colors between illuminants. That is, although if one is asked to judge a color or a stimulus in a controlled setting minimizing context and putting emphasis on light, there may be differences, if they view an object or a depicted image for which individuals ‘know’ the color and are not made aware of the changing light, the images do not tend to look different. Such a phenomenon might obviously tend to minimize impact from light on appreciation of art.

#### Matching Display Lighting to the Intentions/Making Conditions of Artists

Finally, the above issues also touch one other, rarely empirically explored, aspect that will be considered in this paper: artists themselves might have specific recommendations for lighting or display context, or, certain artwork making conditions may assume certain lighting types. Certainly, such arguments are well-documented in art history (e.g., Newhouse, 2005). Authors note the phenomenon of al fresco painting or of artists working in studios with Northern lighting, and suggest that natural lighting (i.e., 5500 K) would be the ideal conditions to view paintings as well (Kemp, 1990; Pinto et al., 2006, 2008; Pridmore, 2017). Other artists may seek out special interactions with lighting via glazes or color palette (Olszewski, 1985; Newhouse, 2005).

To our knowledge, only one study has actually investigated lighting with more specific artist intentions, also highlighting the importance of spontaneous interactions via a between-participants design. Leonards et al. (2007; see also Supplementary Appendix Table A1) assessed perceptions of the Renaissance painter Duccio’s ‘Annunciation,’ which depicts a virgin and angel and made strategic use of gold leaf to highlight symbolically important regions (such as the hand of the virgin). The researchers measured the reflective properties of gold leaf and then created digital versions of the painting under lighting conditions mimicking beeswax candlelight (expected light for the artwork) and contemporary display conditions. Individuals viewed the painting in one or the other condition on a monitor while tracking eye movements. The candlelight group had more fixations on the gold leaf areas, rather than areas of typical saliency such as bright colors or faces. They concluded that gold leaf creates a dramatic glow effect when lit by candles, which would be anticipated by the artist.

This raises an intriguing further possibility for lighting interactions, and especially appraisals, as they occur in a gallery. Note, the above study did not include participant ratings of the artwork.

### Present Study

The present study used a multi-part procedure to begin testing, in an ecologically valid manner, how lighting influences our spontaneous aesthetic experiences of real artworks: The first part of this project involved the background analysis of how existing art museums themselves light their art. This involved sending a researcher into a representative sample of museums within the Vienna area to measure ambient light conditions of gallery spaces. As one of Europe’s preeminent cultural capitals and destinations for art tourists, this provided a large number of museums (containing both classical and contemporary art, although of course confined to only one city). These are briefly reported below in order to provide one more line of information to interested readers regarding the existing range or potential commonalities of lighting approaches. This preliminary research also provided a range of concrete lighting examples for use in the subsequent studies.

In Study 1–2, we then considered if differences in lighting type, selecting from the specific museum examples, modulate both the hedonic ratings (liking, assessed beauty, interest) of art as well as the felt emotional experience and willingness to pay to revisit the works. The studies also made use of both representational (portraits) and abstract paintings, borrowed from area museums and artists. The selected lighting temperatures were chosen to provide a general progression from bluer to yellower (warmer) shades. Thus, although largely exploratory, as a working hypothesis it was expected that we might find either general main effects for certain CCTs (e.g., improvements in both subjective mood and art appraisals as the light temperature moved to the warmer end), or transversely, we might detect an interaction whereby specific temperatures resonated best with specific works or broader abstract/representational styles. We also allowed the same participants to specifically set the light temperature themselves to add one more data point to the above-reviewed range of findings. In Study 2, we further explored the question of whether artworks made by an artist within, or to match, specific lighting conditions, do in fact show a resulting connection to the actual ratings of individuals when shown in the same or different conditions. This was done by using three abstract works painted by an artist with the foreknowledge and actual use of the light apparatus used in our studies, and with each specific work designed to be particularly suited to one lighting level (in regards to its contrasts and colors).

## Background Collection of Ambient Lighting Conditions in Museums

### Method: Stimuli/Materials and Procedure

To create a beginning understanding of lighting conditions as actually used in museums, measurements were made in 15 institutions, selected in order to provide a representative range of more classical and contemporary spaces and art, as well as to account for all of the major and some lesser-known museums in the area. The measurement procedure was developed together with researchers at Graz University of Technology. All selected exhibition spaces, representing the main or most-representative gallery of the museums, had similar rectangular floorplans, thus the same procedure was used in all cases. The researcher used as a measuring tool a digital spectrometer (UPRtek MK350), capable of recording CCT, CRI, and lux, as well as the RGB color space (i.e., CIE1931). To measure the general ambient lighting conditions (illuminance) of the rooms, the digital spectrometer was positioned at a standard height of 155 cm above the floor at the center of each gallery space and facing one of the four corners. The spectrometer measured the light conditions in the form of a sphere, however, only the average of the first quarter, facing the corner, was recorded. The procedure was repeated for each of the four corners, with the four results then averaged. To measure the CRI, a series of measurements of luminance, or reflected light off of the room surface, were taken with the tool 155 cm above floor and 1 meter from both exhibition walls as well as 10 cm in front of the canvas of all paintings in the direction of the light source, with the results averaged into general measures. The spectrometer was re-calibrated before each individual measurement.

### Results and Discussion

Table 1 displays the lighting conditions (specific museum names have been withheld, however a general description of artwork type is provided). As can be seen, the results do suggest quite a range of lighting CCTs—5328 K at the coolest temperature to 2919 at the warmest—covering the spectrum of the arguments in the above literature review. The mean temperature (3759.3 *SD* = 727.9) was generally higher than the earlier Kruithoff-based/museum best practice arguments for around 3000 K, and much closer to the empirical findings of preferred illuminants around 3600 K from light box studies. A comparison based on the broad types of art in the museum collections showed that galleries with classic artworks tended to have the lowest, quite consistent CCTs (*M* = 3274 K, *SD* = 92.0); museums with Pre-Modern (e.g., impressionism, etc.) to early Modern artworks had higher (*M* = 3686.3, *SD* = 802.8), and museums with late Modern to contemporary art had the highest (*M* = 3977.8, *SD* = 789.0).

**TABLE 1:** Ambient lighting conditions in galleries of museums in the Vienna area.

**Museum**	**Art type**	**Date of**	**CCT**	**CRI**	**Lux**
		**measurement**	**(K)**	**(0–100)**	
04	Classic	5/28/17	3175	88	66
11	Classic	3/7/18	3357	92	399
14	Classic	3/30/19	3290	85	135
02	Pre-Modern	4/7/17	4838	87	505
08	Pre-Modern – Modern	11/9/17	3397	91	64
13	Pre-Modern – Modern	3/9/19	3530	93	281
15	Pre-Modern – Modern	5/14/19	2980	90	148
06	Modern – Contemporary	8/12/17	4824	89	126
07	Modern – Contemporary	8/12/17	3618	79	62
12	Modern – Contemporary	3/7/18	4192	89	70
01	Contemporary	3/29/17	3572	90	50
03	Contemporary	4/30/17	2919	95	108
05	Contemporary	10/17/17	5328	86	697
09	Contemporary	11/14/17	3989	96	195
10	Contemporary	11/24/17	3380	81	216

*Values represent an average value from multiple systematic measurements of single exhibition spaces inside the selected institutions. The chosen exhibition space represented the most representative room in the institutions.*

The measured illuminance, overall, also showed a wide range—50 to 697 Lux. Interestingly, while the classic art museums had illuminations directly in line with typical best practice guidelines (*M* = 200 lx), Contemporary galleries actually had a lower mean of 190.5 lx, while Pre- to early-Modern museums showed a higher 249.5 lx. A rather large positive correlation (*r* = 0.617) was found between CCT and Lux. This of course would tend to go against the general Kruithof-based practice, and with these findings suggesting, as intuited in the introduction, that there does appear to be, even within this small sample, a large number of lighting solutions with little in the way of clear shared patterns.

## Study 1 and 2—Empirical Comparison of Lighting Conditions With the Same Art

We then moved to the experimental portion of this project, wherein we considered if lighting, using specific examples chosen from above, modulates the appreciation of art.

### Participants

The studies involved 63 participants (32 female; *M*_age_ = 22.63, *SD* = 2.32), recruited as part of a bachelor’s seminar at the University of Vienna, however on a voluntary-basis without class credit or other remuneration. All participants had normal or corrected-to-normal vision and were not color blind. All were art novices (as confirmed by post-study interview and survey), without any previous training in art history, philosophy, or art production. The gender distribution was intentionally balanced (as much as possible), due to previous suggestion of gender differences in regard to hedonic responses to lighting conditions (e.g., females tend to prefer softer, warmer, less intense light, Knez and Kers, 2000). However, this did not prove to be a key factor in the findings (see also below).

All participants completed both Study 1 and Study 2. However, as described more fully below, the lighting conditions were changed between-participants, with the participant sample therefore further divided into groups based on the specific lighting condition (see also Table 2), leading to 20 participants for Study 1 Condition 1, 20 for Condition 2, and 23 for Condition 3 in the portrait rooms; and 22 participants for Study 2 Condition 1, 21 for Condition 2, and 20 for Condition. Importantly, groups did not show any significant differences in age, gender distribution, or art knowledge.

**TABLE 2:** Lighting conditions for Studies.

**Condition**	**CCT**	**Lux**	**CRI**	**Museum/type (from prelim. Study)**
	**(Kelvin)**			**[‘ideal’ artwork match for Study 2]**
**Study 1 (Portraits)**				
Condition 1	3175	580	93	04/Classic
Condition 2	3572	590	92	01/Contemporary
Condition 3	4838	505	87	02/Modern-Contemporary
**Study 2 (Abstract)**				
Condition 1	3572	590	92	01/Contemporary [Artwork 4]
Condition 2	4838	505	87	02/Modern-Contemporary [Artwork 6]
Condition 3	5328	520	86	05/Contemporary [Artwork 5]

### Materials and Room/Art Set-Up

**FIGURE 2:**
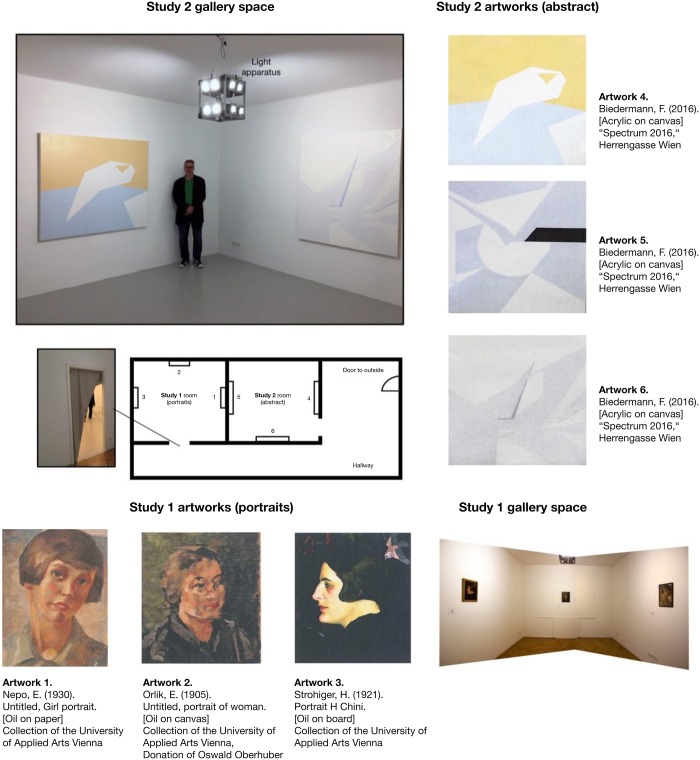
Study location layout and included artworks. Light apparatus is shown in top left. Study 1 (portrait) paintings reprinted with the permission of the copyright holder (Artwork 1: Ernst NEPO, ohne Titel, Mädchenportrait, around 1930, oil on paper, 39 cm × 29 cm. © University of Applied Arts Vienna, Collection and Archive, Inv. No. 4448/B. Artwork 2: Emil ORLIK, ohne Titel, Frauenbildnis, 1905, oil on canvas, 38.5 cm × 35 cm. © University of Applied Arts Vienna, Collection and Archive, Inv. No. 5552/B, Donation by Oswald Oberhuber. Artwork 3: Hans STROHOFER, “Portrait H. Chini,” 1921, oil on board, 35 cm × 31 cm. © University of Applied Arts Vienna, Collection and Archive, Inv. No. 2649/B). Study 2 (abstract paintings) re-printed with the permission of the artist (© Friedrich Biedermann). Photographs of rooms were taken by the authors.

For the studies, two gallery spaces were provided to the authors by the University of Applied Arts Vienna (see Figure 2). Both rooms were 4 × 4 m, with a doorway on one wall to an outside anteroom and without any other views or windows to the outside or to each other. The doorways to both rooms were also covered with a curtain to block any ambient outside lighting. The rooms were painted white (walls and ceiling), mimicking a typical ‘white cube’ gallery. The specific paint for the walls (clear white matte, “StoColor Rapid Ultramatt”) was chosen based on pilot testing (seven samples of different manufacturers, all recommended for exhibition spaces) to maximize the range of wavelengths reflected so as to ensure the fidelity of the artwork colors and lighting. Each room was fit with a light apparatus, hung in the center (see below), and had three different artworks (each hung individually on one wall). The common anteroom was used as a welcome area during the study phase. All windows of the anteroom were also covered and it used a light source with a similar spectrum and CRI to those used inside the exhibition spaces, so that the eyes of the visitors could adjust to the artificial light.

**FIGURE 3:**
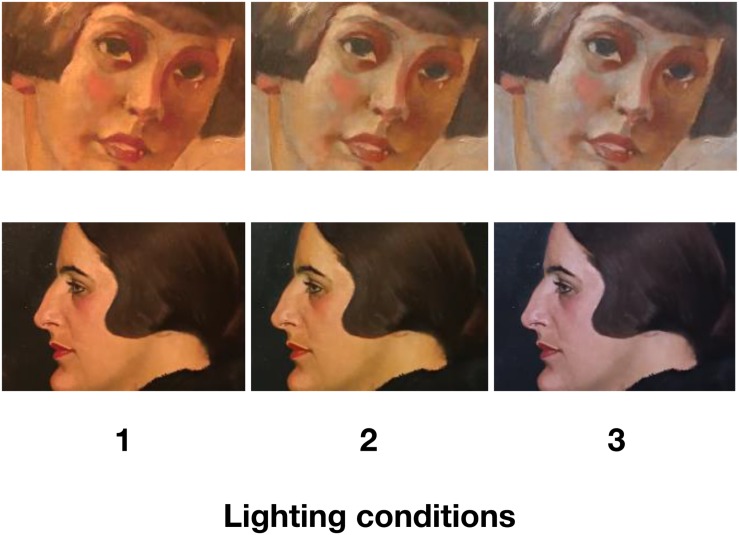
Paintings (Study 1, portraits) seen under different lighting conditions. Paintings reprinted with the permission of the copyright holder (Top: Ernst NEPO, ohne Titel, Mädchenportrait, around 1930, oil on paper, 39 cm × 29 cm. © University of Applied Arts Vienna, Collection and Archive, Inv. No. 4448/B. Bottom: Hans STROHOFER, “Portrait H. Chini,” 1921, oil on board, 35 cm × 31 cm. © University of Applied Arts Vienna, Collection and Archive, Inv. No. 2649/B). Photographs of paintings under different lighting conditions were taken by the authors.

The paintings for Study 1 consisted of three figurative portraits of young women (all similarly sized oil on canvas with a realistic/slightly impressionistic style consistent with the early 20th century; see Figure 2 for images and artist information; Figure 3 shows the paintings under the different lighting conditions) from the collection of the Oskar Kokoschka Zentrum of the University of Applied Arts Vienna. The paintings were selected in agreement with a curator and art historians, included a generally wide-range of colors and darkness/lightness, and represented artworks that we expected most novice viewers might consider to be ‘typical’ non-abstract paintings as seen in many museums.

Study 2’s paintings consisted of three abstract artworks (acrylic on canvas; all 1.4 × 1.6 m; see Figure 2). All were by the artist Friedrich Biedermann (from the 2016 series, “Spectrum 2016”). The paintings consisted of geometric shapes on a colored background. All were painted in such a way as to specifically anticipate a certain lighting condition. This was done by converting the measured light conditions (CIE1931 color space, which provides a measure of the mixture of RGB elements) taken from museums in Study 1 and mixing the actual paint for the artworks so that these matched. Thus, as noted in the introduction, when displayed under the corresponding lighting, the chosen colors would be accentuated or, transversely, if shown under light with opposing characteristics, would become generally black or fade into the background.

### Lighting Apparatus and Conditions

The light source for both rooms was a custom unit created in part for this study (by studio Okular, Mag. AG, Architect). These used a cube-like design with four LED spots (Human Centric Lighting system PiLED, 500 mA with mixing chamber, SMD high power LED Module, Lumitech Produktion und Entwicklung GmbH, Jennersdorf, Austria) on each side of the apparatus. This allowed the control of CCT (1800 to 16,000 K) as well as visible colors (CIE-xy points and RGB colors) and illuminance. For both rooms, the lighting conditions were controlled via a laptop computer (PiLed and Loxone software) situated in a storage space not visible to the participants.

For the purpose of the studies, we used lighting conditions corresponding to actual conditions within three Viennese museums as measured in the preliminary study, manipulating CCT while keeping Lux relatively constant (505–590). Note also that the CRIs were relatively constant as well (86–93). For Study 1 (portrait room), which tested the basic potential for different lighting to modulate appraisal or art experience, we selected three light conditions covering a general range from 3175 K, corresponding to typical Kruithof-based museum conditions; 3572 K, corresponding to suggested CCT findings from lightbox studies; 4838 K, corresponding to monitor and potentially museum-based results (Nascimento and Masuda, 2014). Study 2’s (abstract art) room also used three lighting conditions with a similar range. These also corresponded to actual lighting conditions of specific museums (see Table 1). However again, in this case, these were selected by the artist to specifically match ideal conditions (accentuating blues, yellows, grays, respectively) for enhancing the experience when viewing the paintings.

### Procedure

Participants were invited to the testing location and met in the hallway outside the testing rooms. Participants were informed that they would be asked to view a selection of art and to make some ratings and signed an informed consent. Importantly, no mention was made of the varying light conditions, and participants were only exposed to one lighting condition per room. The participants were led individually to each room, given a paper survey and pencil, and asked to enter, view, and then to rate the individual paintings using the corresponding scales. The participants had no time limit and were asked to treat the encounter as if they were visiting a museum or gallery. After they finished viewing and rating the art in one room, the first survey was collected and the entire procedure was repeated for the second room.

#### Artwork Rating Surveys

The post-viewing surveys for both rooms consisted of a series of Likert-type scales assessing: (1) general artwork appraisal (beautiful-ugly, like-dislike, interesting-boring, would/would not pay to see again; 7-point, bipolar)^2^. These terms were selected to coincide with many previous empirical studies of art and generally assessed aspects of hedonic appraisal as well economic factors of interest to museums. (2) We also assessed general emotional experience when viewing the paintings using three unipolar 7-point scales (1 = ‘not at all’; 7 = ‘extremely’) for positive emotions, negative emotions, and arousal. The scales were repeated three times in each room for the different artworks with a label identifying the artwork they should be addressed to (and corresponding to a wall label).

#### Self-Adjustment of ‘Optimal’ Art-Viewing Light Temperature

Finally, after completing both artwork viewing/rating tasks, participants were asked to again enter the portrait (Study 1) room and to adjust the color temperature (Kelvin) of the lighting using a sliding scale on a provided laptop connected to the LED light. The task used the following directions: “Imagine you are a curator tasked with adjusting the lighting so that the artworks look best.” Before entering, a researcher first entered the room and set the ambient light temperature (bottom, top, or middle of the range, counterbalanced between participants) in order to control for potential anchor effects. After the participant had made their adjustment, the levels were recorded using both the laptop software and matched to a spectrometer reading (UPRtek M350).

#### Ethics Statement

This study was carried out in accordance with the recommendations of the Ethics Committee of the University of Vienna. All subjects gave informed consent in accordance with the Declaration of Helsinki. The protocol was approved by the Ethics Committee of the University of Vienna.

## Results

All participants completed all sections of the study, and all data were used in the following analyses. As noted above, due to previous research suggesting a potential gender difference in response to certain lighting conditions, we first compared responses between male and female respondents. However, independent *t*-tests conducted within each lighting condition showed no significant differences for all scales. The data were therefore combined in the following analyses.

### Study 1 (Portraits): Lighting’s General Impact on Representational Art Experience

**FIGURE 4:**
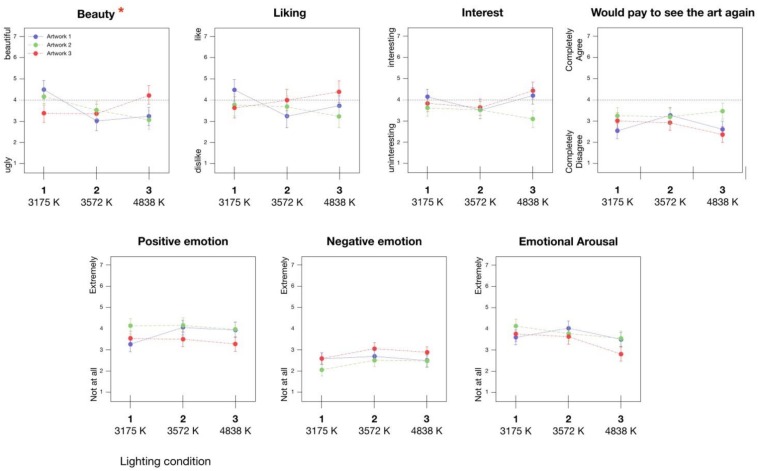
Effect of changing lighting temperatures (Kelvin) on appraisals and felt emotion with representational art (^∗^ corresponds to significant interaction between lighting and paintings, *p* = 0.004, mixed ANOVA lighting × artwork. No significant main effects for lighting were detected for any scale).

Results with artwork appraisals are summarized in Figure 4. As can be seen, appraisals of the artworks tended to fall in a range at about the midpoint of all scales. Answers for the four different rating scales also showed moderately high significant correlations with each other within each artwork (e.g., ratings for Artwork 1 all *r*s = 0.55 to 0.85; Artwork 2 *r* = 0.59 to 0.74; Artwork 3, *r* = 0.27 to 0.82)^3^. Looking to ratings on the same scales between the artworks, made again by the same individuals, both beauty and liking did not show significant correlations, whereas, ratings for interestingness (all participants’ *r*s = 0.22 to 0.43) and willingness to pay (*r*s = 0.50 to 0.47) did show a significant positive correlation.

To analyze our first research question, regarding the impact of lighting on the experience of paintings, we ran a series of repeated measures ANOVAs with the three *Paintings* as a within-participant factor and *Lighting* condition as a between-participant factor. These were conducted for each of our dependent variables (appraisals and general emotion) separately. The results are shown in Tables 3, 4. Note, due to the exploratory nature of this study, we discuss the results below and throughout the paper without correction for multiple comparisons. However, the reader should be mindful of this point when making any inferences. For the reader who is interested in such a correction, we include information on adjusted alphas following Bonferroni correction in the table notes.

**TABLE 3:** Results of ANOVAs for Lighting Conditions × artwork differences in regards to four hedonic appraisals of art (Portraits).

	**F (df)**	**η*_p_^2^***	***p***
**Beauty**			
Lighting	2.608 (2, 59)	0.081	0.082
Painting	0.066 (2, 118)	0.001	0.936
Lighting × Painting	4.092 (4, 118)	0.122	0.004^∗^
**Liking**			
Lighting	0.476 (2, 58)	0.016	0.624
Painting	1.215 (2, 116)	0.021	0.300
Lighting × Painting	2.044 (4, 116)	0.066	0.093
**Interest**			
Lighting	0.528 (2, 59)	0.018	0.593
Painting	3.755 (2, 118)	0.060	0.026^∗^
Lighting × Painting	1.819 (4, 118)	0.058	0.130
**Willingness to pay to see**			
Lighting	0.295 (2, 59)	0.010	0.745
Painting	3.882 (2, 118)	0.062	0.023^∗^
Lighting × Painting	1.641 (4, 118)	0.053	0.168

*Results based on mixed ANOVAs with painting (3) as a within-participants factor, lighting condition (3) varied between participants, and with each of the four appraisals (beauty, liking, interest, willingness to pay) as the dependent variable, conducted separately. ^∗^Denotes significance at *p* < 0.05. All statistics are reported *without* Bonferroni correction for multiple comparisons. Probability values in bold designate items that would retain significance following familywise correction (all individual comparisons = 21, including positive emotion, negative emotion, and emotion arousal from Table 4; adjusted α = 0.0024).*

**TABLE 4:** Results of ANOVAs for Lighting Condition × artwork differences in regards to reported emotional arousal and valence while viewing art.

	**F (df)**	**η*_p_^2^***	***p***
**Emotional arousal**			
Lighting	1.206 (2, 59)	0.039	0.307
Painting	2.347 (2, 118)	0.038	0.100
Lighting × Painting	1.171 (4, 118)	0.038	0.327
**Positive emotion**			
Lighting	0.232 (2, 59)	0.008	0.794
Painting	4.887 (2, 118)	0.076	0.009^∗^
Lighting × Painting	1.161 (4, 118)	0.038	0.332
**Negative emotion**			
Lighting	0.433 (2, 59)	0.014	0.651
Painting	4.391 (2, 118)	0.069	0.014^∗^
Lighting × Painting	0.503 (4, 118)	0.017	0.734

*Results based on mixed ANOVAs with painting (3) as a within-participants factor, lighting condition (3) varied between participants, and with each of the three emotion factors as the dependent variable, conducted separately. ^∗^Denotes significance at *p* < 0.05. All statistics are reported *without* Bonferroni correction for multiple comparisons. Probability values in bold designate items that would retain significance following familywise correction (all individual comparisons = 12; adjusted α = 0.0042).*

As can be seen, we found no significant main effect for *Paintings* in terms of beauty and liking, however, mirroring the correlations above, we did find a significant main effect for interest and willingness to pay. Moving to our main research question, we found no main effect for *Lighting* on any of our rating variables. However, we did detect a significant interaction of *Lighting* × *Paintings* for ratings of beauty. This suggests that different lighting conditions either relatively lowered or raised beauty ratings for specific works of art in different ways depending on the specific painting. Looking at Figure 4, lighting Condition 1 tended to lead to relatively higher beauty ratings for Painting 2 and 1, when compared to the other lighting conditions. On the other hand, this same lighting condition tended to lead to relatively lower beauty ratings for artwork 3, especially when compared to lighting Condition 3. Condition 2, which, incidentally also corresponded to a museum showing contemporary art, tended to coincide with generally low beauty ratings for all three artworks.

Although not significant, it is worth noting that lighting did also show a trend in regards to a main effect on ratings of beauty (*p* = 0.08), while a similar trend regarding the *Lighting* × *Paintings* interaction was also found for liking, and, in conjunction with the beauty finding, suggesting that there might at least be some lighting conditions that are suitable for both particular artworks and for the overall art style (portaits). However, looking to the effect sizes, with the exception of ratings for beauty, very little impact was detected for lighting conditions in the rooms. For comparison, the effect sizes (Table 3) regarding a main effect of the different paintings were from two to six times larger.

The results of analyses regarding emotional experience are shown in Table 4, see also Figure 4. In this case, we again found a main effect of *Paintings* on both positive and negative emotions. Artwork 3 appeared to evoke more negative emotions (and less positive) than Artworks 2 and, to a lesser extent, 1. Both the main effect of *Lighting* and the *Lighting* × *Painting* interaction were not significant.

### Study 2: Lighting and Ratings/Emotional Experience With Abstract Art

**FIGURE 5:**
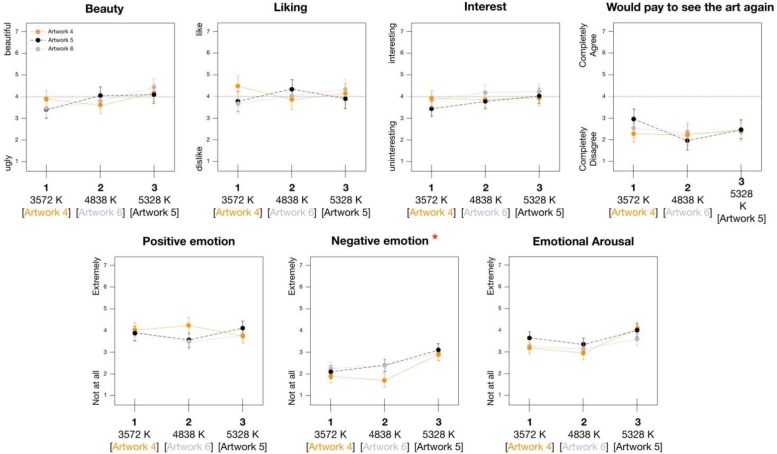
Effect of changing lighting temperatures (Kelvin) on appraisals and felt emotion with abstract art. Color coding of artwork labels corresponds to lighting condition suggested as ideal for viewing by artist (^∗^ corresponds to significant main effect for lighting on rating, *p* = 0.010, mixed ANOVA lighting × artwork).

We then considered the impact of lighting on the abstract art (Figure 5; note, the color coding for the lighting conditions and paintings identifies the artist-intended combinations). In the case of the ratings, once again, the general level of scores was similar to those with the representational portraits in Study 2, falling in a range around the midpoint of all scales. We also again found a correlation of all appraisal scales within each artwork. In addition, individual ratings (e.g., beauty, liking) were significantly correlated between all pairs of artworks (*r* = 0.26 to 0.60), presumably because these were even more similar in terms of style compared to the art from Study 1.

The results of another series of repeated measures ANOVAs with *Paintings* as a within-participants factor and *Lighting* condition as a between participants factor are shown in Table 4 and 5 (see also the table note for information on Bonferroni correction). In this case, no significant effects were found for *Paintings* on any of the appraisals. Similarly, no significant effects were found for *Lighting* or for the *Lighting* × *Painting* interaction, suggesting that not only did specific lighting styles not generally modulate the appraisals of the art, but the artist-intended matches between certain paintings and lighting conditions did not show the expected differences when compared to other, non-intended lighting conditions. The only significant result was a main effect of *Lighting* on negative emotions. As can be seen in Figure 5, this appeared to be driven especially by lighting Condition 3, denoted by a particularly blueish light, which led to higher negative emotion ratings for all three paintings.

**TABLE 5:** Results of ANOVAs for Lighting Conditions × artwork differences in regards to appraisals and reported emotion with Abstract art.

	**F (df)**	**η*_p_^2^***	***p***
**Beauty**			
Lighting	1.878 (2, 60)	0.059	0.162
Painting	0.038 (2, 120)	0.001	0.963
Lighting × Painting	0.963 (4, 120)	0.031	0.431
**Liking**			
Lighting	0.068 (2, 60)	0.002	0.934
Painting	0.239 (2, 120)	0.004	0.788
Lighting × Painting	1.413 (4, 120)	0.045	0.234
**Interest**			
Lighting	0.528 (2, 60)	0.009	0.755
Painting	1.469 (2, 120)	0.024	0.234
Lighting × Painting	0.456 (4, 120)	0.015	0.768
**willingness to pay to see**			
Lighting	0.636 (2, 60)	0.020	0.538
Painting	0.289 (2, 120)	0.005	0.749
Lighting × Painting	1.303 (4, 120)	0.042	0.273
**Emotional arousal**			
Lighting	1.486 (2, 59)	0.048	0.235
Painting	1.923 (2, 118)	0.032	0.151
Lighting × Painting	0.907 (4, 118)	0.030	0.462
**Positive emotion**			
Lighting	0.106 (2, 59)	0.004	0.900
Painting	0.988 (2, 118)	0.016	0.375
Lighting × Painting	1.296 (4, 118)	0.042	0.276
**Negative emotion**			
Lighting	4.929 (2, 59)	0.143	0.010^∗^
Painting	2.878 (2, 118)	0.047	0.060
Lighting × Painting	0.783 (4, 118)	0.026	0.538

*Results based on mixed ANOVAs with painting (3) as a within-participants factor and lighting condition (3) varied between participants, conducted separately for each appraisal/emotion factor. ^∗^Denotes significance at *p* < 0.05. All statistics are reported *without* Bonferroni correction for multiple comparisons. Probability values in bold designate items that would retain significance following familywise correction (all individual comparisons = 12; adjusted α = 0.0042).*

#### Does Lighting Used/Intended by the Artist Show Higher Ratings When Employed in Display?

**FIGURE 6:**
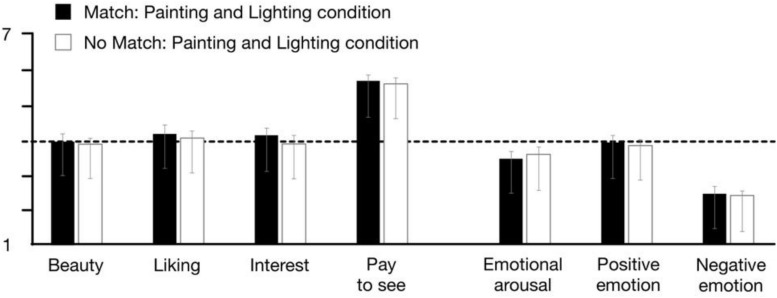
Comparisons of mean ratings and reported emotion when lighting conditions did or did not match the artist-suggested ‘ideal conditions’ for viewing (No sig. differences detected, parallel repeated measures ANOVA, match/no match × Lighting condition/painting group).

Because the artworks had again been designed with the expectation for a specific match to one of the three lighting conditions, we also conducted a simplified analysis in which we compared the responses on the above appraisal and emotion scales regarding the one artwork which was expected to match a specific light condition versus the averaged responses made to the other two artworks which were not expected to match the lighting (see Figure 6). However, again, both a similar series of repeated measures ANOVAs (match/no match × Lighting condition/painting group) as well as *t*-tests across all participants in the differing lighting conditions returned no significant differences or notable trends.

### What ‘Ideal’ Light Temperature Would Participants Choose for Study 1 Art?

Finally, we assessed the results from the last study task in which individuals were asked to set what they would deem the ideal light temperature (in Kelvin, adjusted on a sliding scale from 2715 to 5322) for the Study 1 portraits. An analysis of the results showed that the spectrometer reading and the computer controls consistently provided similar readings. Therefore, as the spectrometer reading taken inside the rooms presumably most closely approximated the actual light temperature as it was perceived by the participant, we used these data for the analyses.

**FIGURE 7:**
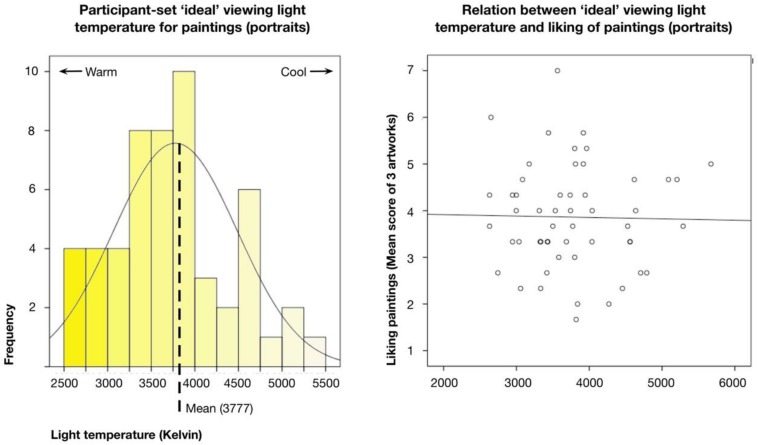
**(Left)** Histogram of participant answers to ‘ideal’ viewing conditions (light temperature in Kelvin) for representational artworks (portraits room, Study 1). Dotted line indicates group Mean. **(Right)** Scatterplot of relation between participants’ settings for ‘ideal’ viewing light temperature of portrait room and liking ratings from previous study of same art.

Figure 7 displays a histogram and group mean of the participant settings for the ‘ideal’ viewing temperatures. Overall, participants showed a mean of 3776.87 K (*SD* = 711.21; Mdn = 3740.50). However, the results also showed a rather wide range of answers between 2631 K at the warm end and 5672 K at the cool end (25th to 75th quartiles = 3323.0 to 4097.25, respectively; highest concentration of answers in 3250 to 3750 bin). Note, the range of participant answers also covered all of the selected museum-based temperature settings, with a mean closest to the portrait Condition 2. Interestingly, in the Study 1 results above, which used the same portrait paintings as used in the present assessment, this lighting condition actually corresponded to one of the only significant effects, regarding lowered beauty ratings. A linear regression with either beauty (*t* = −0.139, *p* = 0.890) or liking (*t* = 0.510, *p* = 0.612) ratings from Study 2 above as dependent variables and the participant settings for lighting temperature as a predictor showed no significant relation. A similar lack of significant results was also found for the other ratings and participant demographic factors, suggesting the absence of a clear relationship between temperature of lighting and appraisal of the art.

## Discussion and Conclusion

We assessed the impact of lighting conditions on the spontaneous appraisal and the felt emotional experience with visual art. This was designed to move beyond previous studies, which often used both non-ecologically valid (non-gallery and real artwork) designs and employed within-participant paradigms with overt emphasis on lighting changes, matched with multiple comparative ratings of the same art or room, most probably inflating emphasis on any lighting influence. In response, we employed, for the first time, a between-participant design allowing us to consider the more spontaneous influence of ambient lighting on works of art as encountered in an ecologically valid gallery setting.

Looking to our results, and considering the titular question for this paper, the most salient takeaway across all of our study components would be an answer of ‘*no, generally lighting did not appear to make much difference to the art experience*.’ In the case of both the representational and abstract paintings, changing the lighting in both gallery spaces did not have any significant main effect on appraisals or, for the most part, emotional experience. Rather, the ratings for the paintings tended to stay within a rather neutral range of scores, moving slightly up and down depending on certain painting-lighting combinations, but well within the error for the studies. Similar results were also found for lighting impact on felt emotional arousal and valence with representational art, and for positive emotions and arousal with abstract. Similarly—and perhaps more surprising—the results from Study 2 suggested that art viewed in conditions different from those in which it was created and certainly not matching those suggested by the artist for best appreciation, also resulted in no detectable difference in the viewer experience.

In fact, the only significant general finding in regards to the experimental questions involved a main effect of lighting on negative felt emotions with the abstract art. As can be seen in the second panel of Figure 4, this appeared to be driven especially by lighting Condition 3, denoted by a particularly blueish light and which was in fact the coolest light setting used in the studies (5328 K). Perhaps more interesting, we also detected an interaction between lighting and paintings for the representational art (Study 1) in terms of ratings of beauty, where lighting Condition 1 tended to lead to relatively higher beauty ratings for Painting 2 and 1, and on the other hand, tended to lead to relatively lower beauty ratings for artwork 3, and lighting Condition 2, which also corresponded to a museum showing contemporary art, coincided with generally low beauty ratings for all three artworks. This indicates of course that there may be combinations of one particular painting with one particular lighting condition that can enhance the aesthetic experience. However, these detected effects should also be considered in light of other contrasting factors. Notably, the differences between paintings themselves, for both abstract and representational art, typically showed two- to six-times the effect size regarding appraisals as did the lighting.

Finally, we also found a rather wide-range when participants were given the chance to set their own ‘ideal’ light temperature for viewing. The Mean temperature (3777 K) roughly coincided with the suggested most enjoyable conditions for everyday living and some past art research (especially the 3600 K as found by Scuello et al. (2004b) in their analysis with postcard art reproductions). This result may lend some credence to Nascimento and Masuda’s (2014) argument that art viewing in actual gallery conditions as opposed to viewing images on a screen, lead to lower (warmer) preferred temperatures. However, it was also lower than the only other previous study conducted with non-reproduction or screen-based works of art (5500 K reported in the Nascimento and Masuda, 2014 study). The differences could of course be due to a number of factors including type of art (in this case representational) which may have better matched a redder or yellower light. At the same time, in our opinion, the more important result was the wide variance—the range of answers went from quite cool to quite warm, covering all of the museum lighting conditions used in our studies, and suggesting that even if individuals prefer a warmer light in general there is again no clear consensus or even pattern to the answers.

These findings, therefore, support a rather—at least to ourselves—surprising conclusion. The actual impact of the lighting as detected in the present study appears to play only a small role in the actual felt emotions and ratings of art. This finding may be key for the curator or art-focused scientist, suggesting that lighting may not really be so important in designing art display. Certainly, there does not appear to be one ideal lighting temperature for viewing art. A similar finding is also found in most of the past lighting research whereby individuals are given the opportunity to select their own CCT. Although there are of course unique one-to-one relationships whereby one lighting temperature may help or harm the reactions to specific works, the effects detected here do not even show the same relationships or directions within an artwork class such as similarly-styled portraits or abstracts pieces by the same artist.

This study also highlights the important methodological difference of within- versus between-participant designs, and suggests that the former, which has been the main form for past lighting and art research, may be driving most effects. This finding would essentially fit the previously suggested results in study designs wherein the lighting changes are obscured from a viewer (e.g., Snow, 1927, of course not considering visual art) and suggesting that if these are not salient, they really do not appear to have much effect. This should be considered or perhaps contrasted within- and between-participant in future lighting and art research.

At the same time, this result also raises the obvious next question: given the persistent emphasis on lighting as a key component of the contemporary gallery, and with lighting’s ability to adjust how an artwork literally looks, why does light not seem to significantly impact art perception or emotional experience? This is important both for the pragmatic question of artwork display and appreciation, as well as for the more general discussion of context in empirical psychological studies of art, which have documented the importance of various modulating factors in appraisal or response.

One explanation might again follow arguments such as a color constancy hypothesis (Foster, 2011; Liu et al., 2013; Nascimento and Masuda, 2014; Berns, 2016; Pridmore, 2017). It may be that viewers, especially with the representational paintings in Study 1, know how a person ‘should’ look or what colors they ‘should be,’ and thus are not that impacted by actual lighting-related changes. In the same vein, the lack of effect found in this study may also relate to, for example, previous studies showing a “facsimile accommodation hypothesis” (Locher et al., 2001, 1999), whereby lab viewers do not always show differences in many types of ratings comparing between original and reproduced or screen-based art (Brieber et al., 2015). People may ‘look past’ the presentation or visual conditions and evaluate the ‘underlying art.’ Such a suggestion is also supported in the original surveys of Kesner (1997) where brightness/contrast were not found to be important factors by art museum visitors. This may be similar to how we approach especially modern or post-modern art which does play with ‘natural’ colors. This result may also be inflated by the lay viewer sample, who may rely more on mimetic content for making ratings (e.g., see Pelowski et al., 2017b; Grüner et al., 2019; for such findings with artwork assessments).

On the other hand, this finding, especially as it regards classic color constancy discussions, is also made more interesting by the inclusion of abstract art. In the case of our selected abstract paintings, these put great emphasis on color, with only minimal geometric design. As these paintings were being seen for the first time, it would be difficult to presume that viewers knew what colors they should be and thus should have focused more on their actual vibrancy or appearance. In the same Kesner (1997) study, visibility and color range were of course also suggested to be key. However, again, when actually tested in our gallery conditions, changing the lighting made no detectable difference. Even more, the artworks and making conditions were in fact chosen by the artist to anticipate certain types of light, which, again, did not show the interaction that was expected. The fact that individuals can look past something so prevalent as lighting, raises interesting questions for future studies of context.

The present study also of course comes with important caveats and demands for future research. The study was confined to only two types of art, with only three examples of each and three lighting conditions. It may well be that a larger study with more viewers and art examples could find lighting-related differences. I should be noted that even relatively tiny effect sizes—such as certain paintings becoming slightly more beautiful—can be meaningful when one considers the sheer amount of people that visit museums. Why would a museum not want to put its art in the best light, if it knows what that would be? Future study might also consider those with more or less interest or knowledge in art, or frequent museum/gallery connoisseurs, for whom we might again detect important differences. The question of how lighting might guide other processes, such as attention (e.g., measured by eye-fixations), may also provide compelling findings. It is our hope that this paper will thus provide an important roadmap to individuals interested in display of art and a useful tool for future research.

## Data Availability Statement

The datasets for this manuscript are not publicly available due to data protection and storage regulations. Requests to access the datasets should be directed to the corresponding author.

## Ethics Statement

The studies involving human participants were reviewed and approved by Ethics Committee of the University of Vienna. The participants provided their written informed consent to participate in this study. Written informed consent was obtained from the individual(s) for the publication of any potentially identifiable images or data included in this article.

## Author Contributions

MP, ES, AG, MF, and HL contributed to the conception and design of the study. ES, MF, and AG contributed to the data collection. MP and ES contributed to the data analysis. MP, ES, JH, MF, and AG contributed to the initial manuscript writing. All authors contributed to manuscript revision, read, and approved the submitted manuscript.

## Conflict of Interest:

The lighting apparatus used in this study was designed by the commercial architecture firm Studio Okular, Vienna, Austria, of which AG is a principle member. However, this entity did not have any relation to the study design, analyses, or interpretations. The remaining authors declare that the research was conducted in the absence of any commercial or financial relationships that could be construed as a potential conflict of interest.

## Footnotes

1 In measuring brightness, a distinction should also be made between “illuminance”—the amount of light that is emitted from a source—and “luminance” or the amount of light that reflects off of a target surface such as a painting (Veitch and McColl, 2001). Many previous studies have involved recordings of the first “illuminance” measure. However, luminance may actually pertain more to lighting’s interaction with art as it is perceived by a viewer, and is thus both important to consider and may give a metric for comparing conditions.

2 Note, the original study design had included four additional scales (active-passive, high quality-low quality, meaningful-meaningless, strongly affecting-weak-affect). However, following data-collection and participant exit interviews, it was determined that these scales were highly redundant with the above selected terms or (in the case of quality) showed wide variance in participants’ interpretation of their meaning. Thus, these were not analyzed or included in the study.

3 Despite the generally moderate to high correlations between the four rating scales, the decision was made not to employ data reduction measures (i.e., Factor Analysis or Principle Component Analysis) due to the smallish size of the sample and our interest in considering what, if any, effects lighting might have on this range of scales of typical interest in previous empirical art research and not on the nature of artwork assessment (i.e., specific axes or clustering of appraisals) itself.
