# Supplementary material for: Does Gallery Lighting Really Have an Impact on Appreciation of Art? An Ecologically Valid Study of Lighting Changes and the Assessment and Emotional Experience With Representational and Abstract Paintings
Source: Front Psychol. 2019 Oct 4;10:2148. doi: 10.3389/fpsyg.2019.02148 (PMC6788350; doi:10.3389/fpsyg.2019.02148)
Supplement: Supplementary file 1 [file Data_Sheet_1.pdf]

Supplementary Appendix

Does Gallery Lighting Really Have an Impact on Appreciation of Art? An Ecologically Valid Study of Lighting Changes and the Assessment and Emotional Experience With Representational and Abstract Paintings

Pelowski, Graser, Specker, Forster, von Hinüber, & Leder

Table A1.
Previous studies on lighting and visual art

Authors	Main focus/elements	Method	Findings
**1. Survey/Comparison of different Museum lighting conditions**			
Kesner (1997)	• Survey.<br>• Comparison of relative importance of differing lighting factors between museum decision-makers (art and non-art museums) and art museum visitors.	Provided list of 21 factors regarding gallery lighting to final sample of n = 130 (response rate = 37%) "museum decision-makers" (conservators, curators, directors, exhibition or lighting designers) at multiple U.S. museums (art and natural history) and rated by asking how much effort and money should be invested toward each factor to achieve the best result. Matched to a similar survey given to visitors (427) within 18 U.S. museums of art.	Groups had different expectations. For museum staff, preservation most important factor; reducing glare or visual comfort were least important. For viewers, art appearance important (visibility, color range, and attractiveness), but also reducing glare and brightness/contrast were least important.
Wilson (2006)	•Survey/Comparison of degree of contrast between object and background in archeological museums and visitor estimates of lighting quality.<br>• Original stone sculptures in original museum spaces.	Short mention in text on different museum lighting conditions. During Joule 'Delphi' project, illuminance contrast (proxy for "normal luminance (brightness) contrast") of art/historical objects (mostly stone sculptures) measured against background in "a variety of archaeological museums in the Mediterranean region" (possibly 12, see Fig. 5), "compared with visitor estimation of lighting quality" (method and N, unknown).	Lighting quality judged by degree of contrast between object and background. Found in both directions (either bright object and dark wall, or vice versa). In some configurations, detail or clarity of object diminished, but "visitors are unaware of this loss of detail". No relation found between illumination and estimated quality.
**2. Self-selection of preferred lighting conditions**			
Scuello, Abramov, Gordon, & Weintraub (2004b)	• Within-Participant.<br>• Two-alternative forced-choice task. Comparison of CCTs for painting appearance (relative preference).<br>• Reproduced artwork miniatures in lightboxes.	Participants (N = 9) observed 4 paintings (postcard-sized reproductions; impressionist/representational depictions of boats on water or women sitting in garden; divided among differing main colors, i.e., blues or reds) inside light boxes painted matte grey and by looking through porthole. For each trial, observed same painting with 2 of 11 light conditions (CCT = 2500-7000 K; illumination constant at about 200-250 lux); selected which they preferred ("whether the painting looked better" and by how much), using 6-point scale. Also varied prior lighting (acclimated by looking into empty light box with different CCT/illumination combinations) to replicate what might occur when an individual enters a gallery from a differently lit room. Repeated for all paintings, combinations of illuminants, and room orders. Each painting seen 20 times under a given illuminant.	General preference for 3600 K (highest % cases selected against alternative). However, results "not overwhelming". 3600K temperature only about 2 points preferred on 6-point scale. Second spike of CCT preference at about 5400 K; 3200 and 5000 K generally highest lack of preference. General consistency across lighting/painting color interactions, however, paintings described as more blue received higher preference scores under higher color temperatures; red paintings showed the opposite trend. Different pre-trial lighting had no effect. "Pronounced individual differences" (not further investigated).
Pinto, Linhares, Carvalhal, & Nascimento (2006)	• Within-Participant.<br>• Two-alternative	Participants (N = 5) viewed 5 paintings on monitor (based on hyperspectral images of Renaissance oil paintings on wood from Museum Nogueira da Silva,	Across all but one painting-light combination, participants generally preferred higher CCT ('cooler' color). Preferred light also revealed

	forced-choice task. Comparison of CCTs for relative preference.<br>• Screen-based reproductions of artwork with computer-generated lighting effect.	Portugal; all portraits of Madonna and child with dark background) under 5 computer-generated CCTs (2856, 3000, 4450, 4874, 6500 K; illumination M = 330 lux, range 200–400). For each trial, same painting shown twice in sequence (5 s each) with two different lightings; participants asked to choose which they preferred. Each lighting-art pair observed 20 times (total 500 trials; 100 evaluations made for each individual painting). Also estimated chromatic diversity (number of discernible colors) for each light-CCT combination.	relatively more colors. The 1 pairing that had not shown clear preference difference also had very similar chromatic diversity.
Pinto, Linhares, & Nascimento (2008)<br><br>(Follow-up/extension of Pinto et al., 2006)	• Within-Participant.<br>• Self-selection of CCT for ideal artwork viewing.<br>• Screen-based reproductions of artwork with computer-generated lighting effect.	Participants (N = 80, split between art-novice undergraduate students participating in laboratory and art museum visitors using computer within course of museum visit) viewed 11 paintings on monitor (oil paintings from same museum; 7 Renaissance representational portraits with virgin and child on wood; 4 still-life or domestic scenes from 20th c. on canvas), under computer-generated CCTs (21 equal steps from 3600 to 25,000 K; illumination M = 330 lux). For each trial, viewed one painting and asked to adjust CCT "for your best appreciation." Also used different background color conditions (black, grey). Lab participants assessed each painting 3 times (conducted on different days); museum visitors 1 time. Also estimated chromatic diversity for each painting-CCT combination.	Different peaks found for each painting. Generally most preferred CCT = 5100 K. Results did not depend on undergraduate students or museum visitors or on background color. Suggested that results go against Kruithof-based expectation for warmer CCTs used in museums and also that "a single CCT [may] be suitable for the set of paintings tested here." Linear regression with chromatic diversity suggested that this could be involved in determining observer's preference.
Nascimento & Masuda (2014)<br><br>(Follow-up/extension of Pinto et al. 2006, 2008)	• Within-Participant.<br>• Self-selection of CCT for ideal artwork viewing.<br>• Screen-based reproductions of artwork with computer-generated lighting effect AND matched real artwork in 'gallery-like' setting.	Participants (N = 7, one familiar with actual research target) viewed 11 original paintings on monitor (same as Pinto et al., 2008) in gallery-like setting. In part 1, for one trial, first viewed each painting on monitor as above, selecting ideal CCT (3600–20,000 K; 200 lux; starting CCT randomized; "select the illuminant such that the appearance of the painting was the most pleasant"), with entire painting set shown twice in random order. Followed by short break and, Part 2, with viewing of actual painting, hung on a wall, each shown individually with adjustable lighting (similar range and preference procedure, spectrally tunable light source, DLP technology OL 490 Agile Light Source). Each painting/CCT adjusted 12 times, presumably in succession, with random CCT starting point set each time before replacing with another real painting. Entire task, with different real paintings each day, repeated on four days (total number of ratings for each artwork in monitor condition = 8; real condition = 12). To ensure even light coverage, in real condition, paintings partially covered with 21 × 30 cm black frame. No mention of whether participants stood/seated, nor if this approximated in any other way an actual gallery.	Both conditions returned similar results, with an average preferred CCT of 5500 for real and 5700 K for monitor (only four paintings differed significantly in posthoc comparisons). Reported that the selected average CCT varied between paintings—with roughly half having a mean CCT lower than the previous study's finding (around 4900-5100) and half higher (6000 to around 8000K), and also varied markedly between participants.

Liu, Tuzikas, Žukauskas, Vaicekauskas, Vitta, & Shur (2013)	• Within-Participant.<br>• Self-selection of CCT/color saturation balance for ideal artwork (diffent content familiarity) viewing.<br>• Real artwork in lightbox. Investigation of potential cultural differences.	Participants (N = 205; split between Chinese and United States citizens) viewed 3 paintings (expected to differ in relative cultural familiarity of content: 1, impressionistic scene with reddish bonfire and women singing with greenish skin tones, acrylic on canvas, expected to be generally familiar for all participants; 2, painting on carboard of Saint George fighting a dragon, employing Middle Ages-esque style in faded blues, reds, greens, and golds, expected to be more familiar for Westerners; 3, post-impressionistic watercolor painting displaying street in Vilnius, Lithuania, with similar colors to painting 1, and expected by the authors to be unfamiliar to all subjects, although, note that the scene itself with buildings, street, and sky is rather universally common). For one trial, viewed art while seated and looking into light cabinet (50 x 50 cm opening), tuned CCT (21 steps, 2500 to 7500 K; 300 lux), as well as the relative balance of AGB versus RGB (impacting color saturation). Asked to choose lighting that resulted in highest subjective appeal of the paintings. These were also compared against a similar study using familiar fruits and vegetables (real objects) of different dominant colors (with different lighting choices).	Lighting preference differed depending on artwork and perceiver, as well culture: Painting 1 = preferred CCT of 2900 for Americans, 3250 K for Chinese. Painting 2 = 3920/3900 (US/Chinese); Painting 3 = 5950/4260. Chinese participants tended to prefer warmer (lower) CCTs and less saturated colors. Painting 3 also showed higher difference between Chinese and Americans, which authors attributed to differing reactions to unfamiliarity of the subject. For familiar natural fruit, no cultural differences.

3. Scale-based Rating of different lighting-art combinations

Scuello (2004a)<br><br>(Follow-up/extension of Scuello et al., 2004b)	• Within-Participant.<br>• Scale-based rating of 8 CCT x painting combinations for painting appearance, clarity, solidity, and color.<br>• Reproduced artwork miniatures in lightboxes.	Participants (N = 9, 2 also participated above) viewed same 4 paintings plus 8 more (12 total; all figurative still-lifes, landscapes, or portraits) within same light boxes under 8 CCTs (2500-6500 K). For each trial, evaluated same painting 8 times in succession under each of the lighting conditions (order randomized); after each viewing, assessed light/painting combination with 11-point scales for: "How much do you like the appearance of this picture under this light?", "How vivid do the colors appear?", "How solid and three-dimensional does the subject matter appear?", "How clear does this picture appear?".	Generally highest ratings for 3600 K. However, effect not pronounced, with lighting in range of 5500 – 6500 K also showing relatively higher ratings. No CCT clearly disliked. Suggested (followed up in Scuello et al. 2004b) that the selection of around 3700K may be tied to general neutrality between perceived warmth and coolness.
Luo, Chou, Chen, & Luo (2013)	• Within-Participant.<br>• Scale-based rating of 15 CCT/illumination x painting (oil, watercolor, Eastern style) combinations for impact on physical/visual attributes and "psychological perception [of] paintings".<br>• Hand painted copies in full-size lightbox room.	Participants (N = 30; half science and engineering students, half art and design students) viewed 6 paintings (copies of original pieces from Taipei Fine Arts Museum, hand-produced by other artists; two portraits of women, one still life, three landscapes; in oil, watercolor, or Eastern painting style), illuminated by 16 LED lights in 15 combinations of CCT (2700, 3500, 4000, 5000, 6500 K) and illumination (50, 150, 300 lux), placed in light cabinet (viewed while seated in front, no other information on hanging aspects). For one trial, viewed one artwork under each lighting combination and made repeated ratings for physical attributes (colorful/dull, bright/dark, clear/blurry) and "psychological perception [of] paintings" (warm/cold, relaxed/tense, soft/hard, natural/unnatural, active/passive, comfortable/uncomfortable, modern/classical, pleasant/unpleasant). Total ratings = 15 times per painting.	Preferred lighting of art and design students was 5000K at 300 lux; science and engineering students was 4000K at 300 lux. Males preferred 4000K, females 5000K (both again at 300 lux). PCA of different appraisal scales suggested two components— "warmth" (warm/ cool but also classical/modern, soft/hard, and presumably connected to CCT) and what they term "visibility" (all other scales, including pleasantness).

Feltrin, Leccese, Hanselaer, & Smet (2017)	• Within-Participant.<br>• Scale-based rating of 5 CCT x painting (different predominant colors) combinations for overall appreciation and color warmth, vividness, brightness, attractiveness.<br>• Reproductions on canvas in full-size lightbox room.	Participants (N = 25; 16 naïve to the study; 9 fellow researchers in the same laboratory) viewed 5 painting reproductions (impressionist, 2 forest paths, a tree lined street in the rain, boats off shore at night; printed on canvas; with either prominent color of red, blue, green, yellow, or combination), placed on metal stand at typical hanging height, while seated in chair 1.4 m away looking into display space (similar to Scuello et al., 2004a). Illuminated by 5 CCTs (3000, 3500, 4000, 5000, 6000 K from tunable LED spotlight in combination with fluorescent ambient light with CCT of 6500 K; CRI of 93; illumination = about 160 lux). Painting backed by 3 curtains (white, grey, black). For one trial, viewed each painting under all light conditions shown successively in random order (all five light configurations shown first, in order to give participants an idea of the lighting differences); rated each combination using 6 bipolar 11-point scales (perceived painting color warmth; color vividness; color brightness; color attractiveness; background color appreciation; overall appreciation of arrangement). Repeated for different backgrounds. Total ratings = 15 times per painting.	CCTs of 3500, 4000, and 5000 K nearly equally preferred, slight peak at 4000; 3000 and 6000 K less liked. Preferences similar for every background (much like Pinto, Linhares & Nascimento, 2008). Similar trend found for all paintings, regardless of predominant artwork hue (generally similar to Scuello et al., 2004a).

4. Consideration of viewer interaction and appreciation in original or approximated gallery setting

Yoshizawa, Fujiwara, & Miyashita (2013)<br><br>(see also Zhai et al., 2015 for discussion and presentation of analyses)	• Within-Participant.<br>• Scale-based rating of 52 CCT/illuminance/CRI x painting combinations (Study 1) and 9 CCT x painting combinations (Study 2) for preference and lighting/appearance factors.<br>• Reproductions in mock gallery/ original art in original museum setting.	Participants (N = ?) viewed 3 painting representations (16th century portrait, 19th century impressionist landscape, 20th century abstract; oil) in mockup gallery space (wood floor with whit walls) under 52 lighting conditions with 4 CCTs (2700, 3000, 4000, 5000 K), 4 illuminances (50, 100, 200, 400 lux), 3 CRIs (95, 90, 59); LED lighting. Also conducted second study in Morohashi Museum of Modern Art, Japan with real oil paintings (?) under 9 LED conditions (CCTs from 2900 to 4700 K) and one halogen lamp with a diffusion filter (CCT 3050 K; all illuminance = 150 lux). For each trial, view each painting-lighting combination and evaluate using bipolar scales (colorful-drab; easy-difficult clarity; too much-not enough light on paintings; exhilarating-depressing; warm-cool (Study 1 only); moist-dry; preferable-not-preferable; contrasty-not contrasty; glossy-not glossy, deep-flat; rich-not rich in texture.	Ratings assessed with Structural Equation Models (see Zhai et al., 2015); suggested two factors of "Visibility" and "Texture" most important for driving preference for conditions. CCT had a negative correlation with texture and a positive correlation with visibility; illuminance had positive correlation with both factors. CCT showed strongest driver of both factors. Color rendering showed very low relation to variance.

Zhai, Luo, & Liu (2015)	• Within-Participant.<br>• Scale-based rating of 12 CCT/illuminance x painting combinations appearance and for preference and general mood or "atmosphere".<br>• Original oil painting in mock gallery.	Participants (N = 24, split into students majoring in science/engineering and in design/art) viewed 6 original paintings (4 oil/2 gouache, all representational with slight impressionistic style; portrait of man, women, landscape of water and trees with buildings; 3 still life with fruit and flowers; all muted pinks, oranges, and blues) in mock gallery (white walls and ceiling; wood flooring, full room, hung on one wall), under 12 lighting combinations (16-channel LED) of 4 CCT (2850, 4000, 5000, 6500 K) and 3 illuminance (50, 200, 800 lux; lowest and highest corresponding to limits of recommended lighting in museums, CIE, 2004). Notably, LED was directed spotlight, rather than lighting entire room evenly. For one trial, enter and wait for one minute to adapt to lighting, then view one painting under all conditions and made ratings on 14 scales (8-point): "appearance" (Warm-Cool, Bright-Dark, Clear-Unclear, Colorful-Dull, Natural-Artificial); 8 "atmosphere" (High-Low Quality, Active- Negative, Relaxed-Tense, Soft-Hard, Artistic-Business, Lively-Boring, Comfortable- Uncomfortable, Pleasant-Unpleasant). Order of lighting, paintings, and scales randomized (over three sessions taking about 3 hours total). No mention is made of whether the relation of the scales to the painting appearance or to room "atmosphere" (suggested by authors) was actually communicated to the participants.	For more artistic or mood-related scales (Relaxed, Warm, Soft, Artistic), participants' mean ratings decreased as CCT increased (becoming cooler); highest preference at 2850 K.<br>Ratings for contrast, brightness, clarity, and quality peaked around 5000 K. For illuminance, most scales showed positive correlation, tending to peak and then plateau at 200 lux.<br><br>Also conducted PCA and Structure model, showed clusters involving: (1) clarity, (2) warmth, brightness, contrast, (3) comfort or pleasantness, (4) artistic aspects or impression from the art. Artistic ratings decreased over 50 lux.<br><br>Suggested that results "implie[d] that different paintings could be enhanced by applying different lighting conditions," although no further discussion.
Balocco, Farini, Baldanzi, & Volante (2018)	• Within-Participant.<br>• Three-alternative forced-choice task. Comparison of CCTs for preference of painting appearance. Mobile eye-tracking.<br>• Original art (fresco) in original gallery setting.	Participants (N = 15) viewed 1 artwork (wall-sized fresco, "Boscherecce," in Villa La Quiete, Florence; room size = 6.8 x 8.9 m with 4+ m ceiling; depicting a tree-lined path moving toward a building in the background with gardens, statues, and architectural elements in the foreground) under 3 CCTs installed in an antique chandelier (reddish colored, 3047 K; 4049 K light blue/indigo; a LED Standard balanced color, 3782K; also with constant overhead light from 9 LED, ZafiroLED; 70 lux; 2700 K). For a trial, entered room, stood 1.5 m from art, and viewed artwork under each lighting in sequence for 15s each, "expressing your own preference for the fresco, as the selected light scenography changes". Then selected which light preferred; entire paradigm repeated 3 times (no mention of balancing or randomization). Also employed mobile eye-tracking.	54% of participants preferred coolest CCT (blue/indigo, 4049 K). Generally similar looking patterns (areas of interest and visual pathways) across lighting conditions. However, blue light was associated with visual scanning patterns with many fixations oriented to an AOI before moving to another, and lower transition entropy (higher information content transmission). Suggested might tie to higher clarity of colors and brightness (related by authors to Zhai et al., 2015).
**Comparison of original making/viewing light conditions versus alternative**			
Leonards, Baddeley, Gilchrist, Troscianko, Ledda, & Williamson (2007)	• Between-participant<br>• Eye tracking comparison of art perception in two conditions--original making/viewing or contemporary museum display conditions.<br>• Monitor-based computer representation.	Participants (74, split into two conditions) viewed 1 painting (digital reproduction, Duccio's "Annunciation"; Renaissance style; depicts virgin and angel, made with strategic use of gold leaf to highlight symbolically-important regions (e.g., hand of the virgin)) under 2 lighting conditions mimicking beeswax candlelight (proposed original viewing/making conditions) and daylight illumination (contemporary viewing conditions; both computer generated based on assessment of reflective properties of gold leaf). For one trial, view painting on monitor while eye movements tracked.	Candlelight group had more eye fixations on gold leaf areas, rather than on typical areas of saliency (bright colors, faces). Concluded that gold leaf creates a dramatic glow effect when lit by candles, which would certainly be anticipated by the artist, diminished under typical contemporary lighting.
